# Posterior capsule rupture with complete lens dislocation into the vitreous cavity caused by blunt trauma: a case report

**DOI:** 10.3389/fmed.2026.1872348

**Published:** 2026-06-18

**Authors:** Ru Yang, Yuan Tao, Ning Zhang, Xing Du, Yuanyuan Guo

**Affiliations:** Department of Ophthalmology, Jinan Second People’s Hospital, Jinan, Shandong, China

**Keywords:** blunt trauma, lens dislocation, optic disc pallor, posterior capsule rupture, vitrectomy

## Abstract

**Background:**

Complete posterior lens dislocation secondary to posterior capsule rupture following blunt trauma is an extremely rare condition that is easily missed in clinical practice. Here we report a rare case in a 90-year-old patient and summarize its imaging characteristics and surgical management.

**Case presentation:**

A 90-year-old female presented with 20 days of decreased visual acuity in the right eye after blunt trauma. Examinations showed an intact anterior capsule with posterior capsule rupture, complete dislocation of the lens cortex and nucleus into the vitreous cavity, zonular dehiscence, and optic disc pallor of unclear etiology. The patient underwent pars plana vitrectomy, lensectomy, and scleral-fixated intraocular lens (IOL) suspension. Postoperative visual acuity improved gradually, intraocular pressure (IOP) remained stable, and the IOL was well-centered.

**Conclusion:**

Posterior capsule rupture with complete posterior lens dislocation is a rare ocular trauma. Multimodal imaging aids in definitive diagnosis, and individualized minimally invasive surgery is safe and effective. Special attention should be paid to concomitant occult optic neuropathy in elderly patients.

## Introduction

Blunt ocular trauma is a leading cause of monocular visual impairment and blindness worldwide (1). Lens injuries include traumatic cataract, subluxation, and dislocation. Among these, posterior capsule rupture with an intact anterior capsule is extremely rare. Cases with complete posterior dislocation of the entire lens material into the vitreous cavity are even rarer and pose substantial diagnostic and therapeutic challenges.

We present a case of anterior capsule-sparing posterior capsule rupture with complete lens dislocation in a 90-year-old female after blunt trauma. Combined with a literature review, we analyze the underlying mechanism, diagnostic value of multimodal imaging, and selection of individualized surgical strategies, to provide a reference for the clinical management of similar rare ocular traumas.

### Patient information and clinical findings

A 90-year-old female was examined on April 14, 2026, with a 20-day history of decreased visual acuity in the right eye after blunt injury caused by her own fist. Immediate ocular pain, redness and vision loss occurred following the trauma. The patient did not seek medical care timely. Ocular pain and redness subsided gradually, whereas blurred vision persisted, and the patient presented to the ophthalmology outpatient clinic of our hospital.

**Figure 1:**
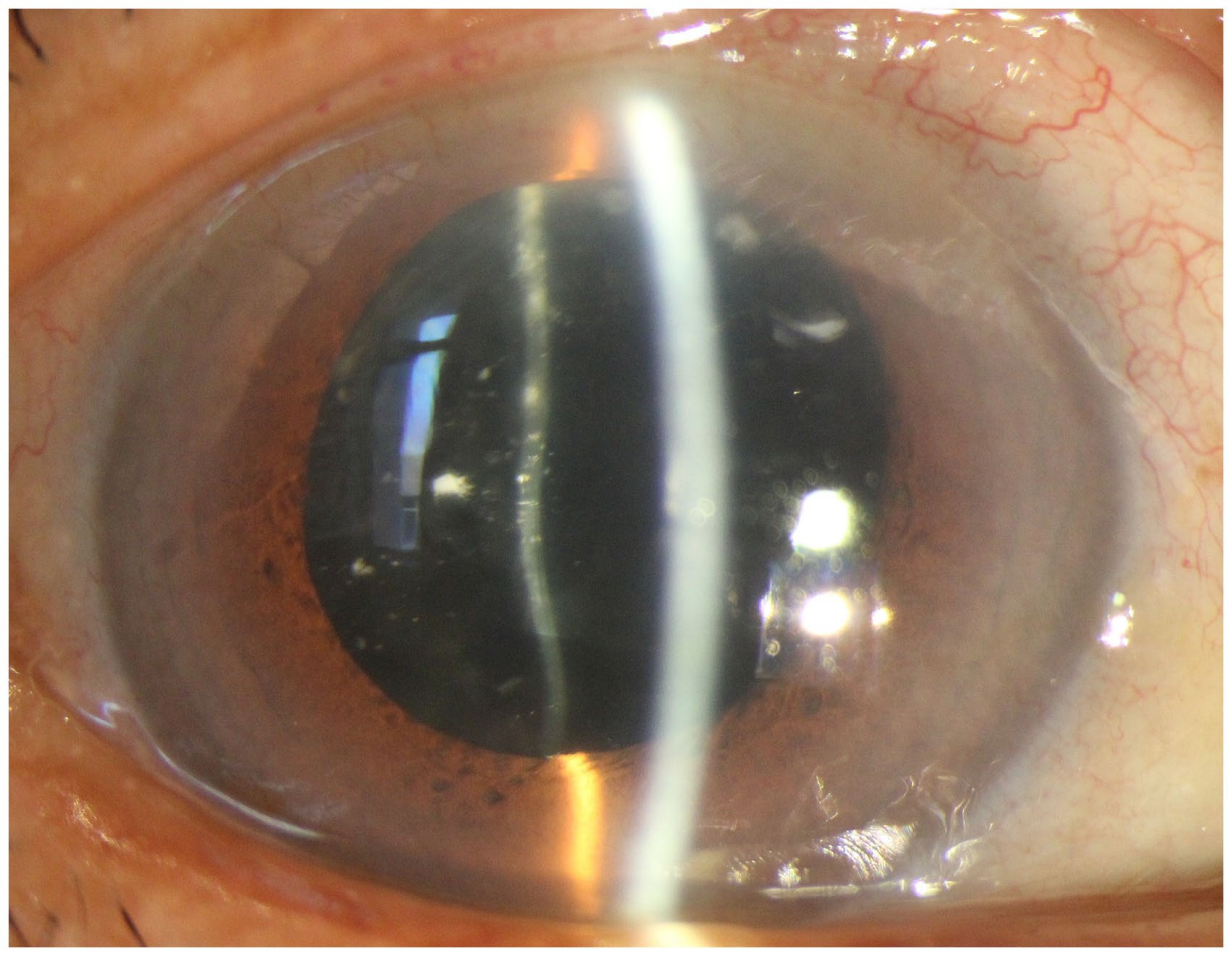
Preoperative anterior segment photograph showing an intact anterior capsule.

**Figure 2:**
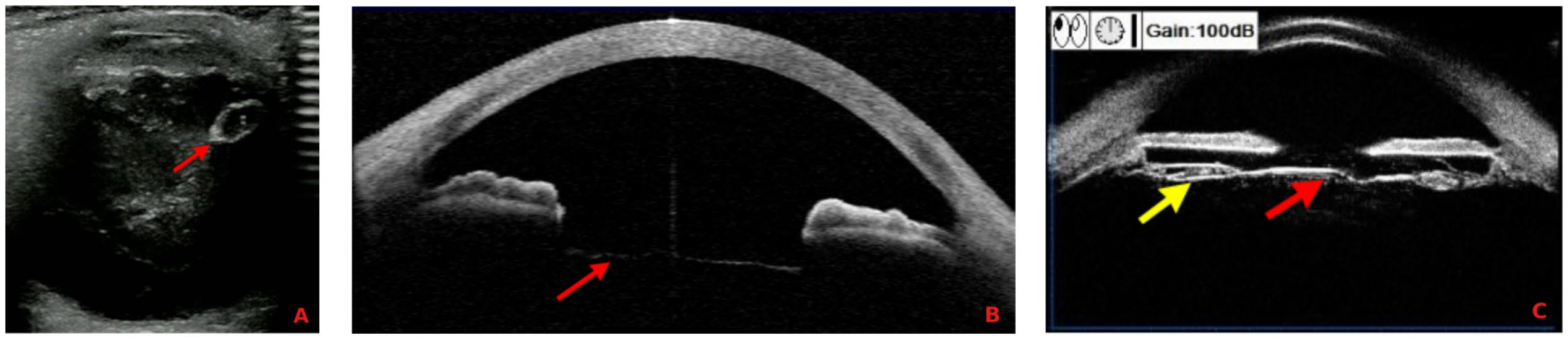
**(A)** Preoperative B-scan ultrasonography of the right eye. The lens nucleus is indicated by the red arrow. **(B)** Preoperative AS-OCT of the right eye. The intact anterior capsule is indicated by the red arrow. **(C)** Preoperative UBM of the right eye. The anterior capsule is indicated by the red arrow, and the ruptured posterior capsule is indicated by the yellow arrow.

The patient was otherwise healthy with no family history of hereditary eye diseases. Best-corrected visual acuity (BCVA) was hand motion in the right eye and 0.6 in the left eye. Intraocular pressure (IOP) was 17 mmHg in the right eye and 16 mmHg in the left eye. Slit-lamp examination revealed mild conjunctival congestion and mild corneal edema in the right eye. The anterior chamber was deepened and quiet, with clear aqueous humor, and no vitreous was found in the anterior chamber The pupil was round, 4 mm in diameter and slightly sluggish to light response. After pupillary dilation, the anterior capsule was intact with no residual cortex or nucleus in the capsular bag (Figure 1). However, pupillary dilation was incomplete, limiting the view of the peripheral zonular region. The vitreous cavity was diffusely hazy, with a yellowish lens nucleus vaguely visible inferiorly, obscuring fundus visualization. The left eye presented clear cornea and normal anterior chamber, with a 3-mm round pupil and normal light reaction. A well-positioned intraocular lens (IOL) was observed without other abnormal findings. Further evaluations of the right eye: B-scan ultrasonography showed vitreous opacity with an oval echogenic lesion, and no retinal detachment was detected (Figure 2A). Anterior segment optical coherence tomography (AS-OCT) confirmed an intact anterior capsule with no lens material within the capsular bag (Figure 2B). Ultrasound biomicroscopy (UBM) demonstrated open anterior chamber angle, without angle recession, intact anterior capsule, ruptured posterior capsule, and asymmetric zonular attachment with variable distances from the zonule to the lens equator, particularly widening in the inferior quadrant (Figure 2C). Diagnoses:1. Complete lens dislocation, right eye2. Blunt ocular trauma, right eye 3 Pseudophakia, left eye.

### Therapeutic interventions

On April 15, 2026, pars plana vitrectomy (PPV) combined with lensectomy was performed on the right eye under local anesthesia. Intraoperatively, the anterior lens capsule was intact with an irregular posterior capsule rupture. Abundant liquefied grayish-white cortex and a brownish-yellow lens nucleus were completely dislocated into the vitreous cavity. The optic disc was pale, and no obvious abnormality was detected in the remaining retina. Zonular dehiscence occurred from 6 to 9 o’clock. Intraoperatively, the remaining zonular fibers, though not frankly ruptured, appeared markedly lax and attenuated, providing insufficient support for stable sulcus IOL fixation. The anterior capsule was removed, and scleral-fixated IOL suspension surgery was conducted subsequently. Postoperatively, the patient received moxifloxacin eye drops (Vigamox, Alcon, USA) four times daily for 4 weeks, 1% prednisolone acetate eye drops (Pred Forte, Allergan, USA) four times daily for 4 weeks, and compound tropicamide eye drops (Mydrin-P, Santen, Japan) once nightly for 1 week.

### Follow-up and outcomes

**Figure 3:**
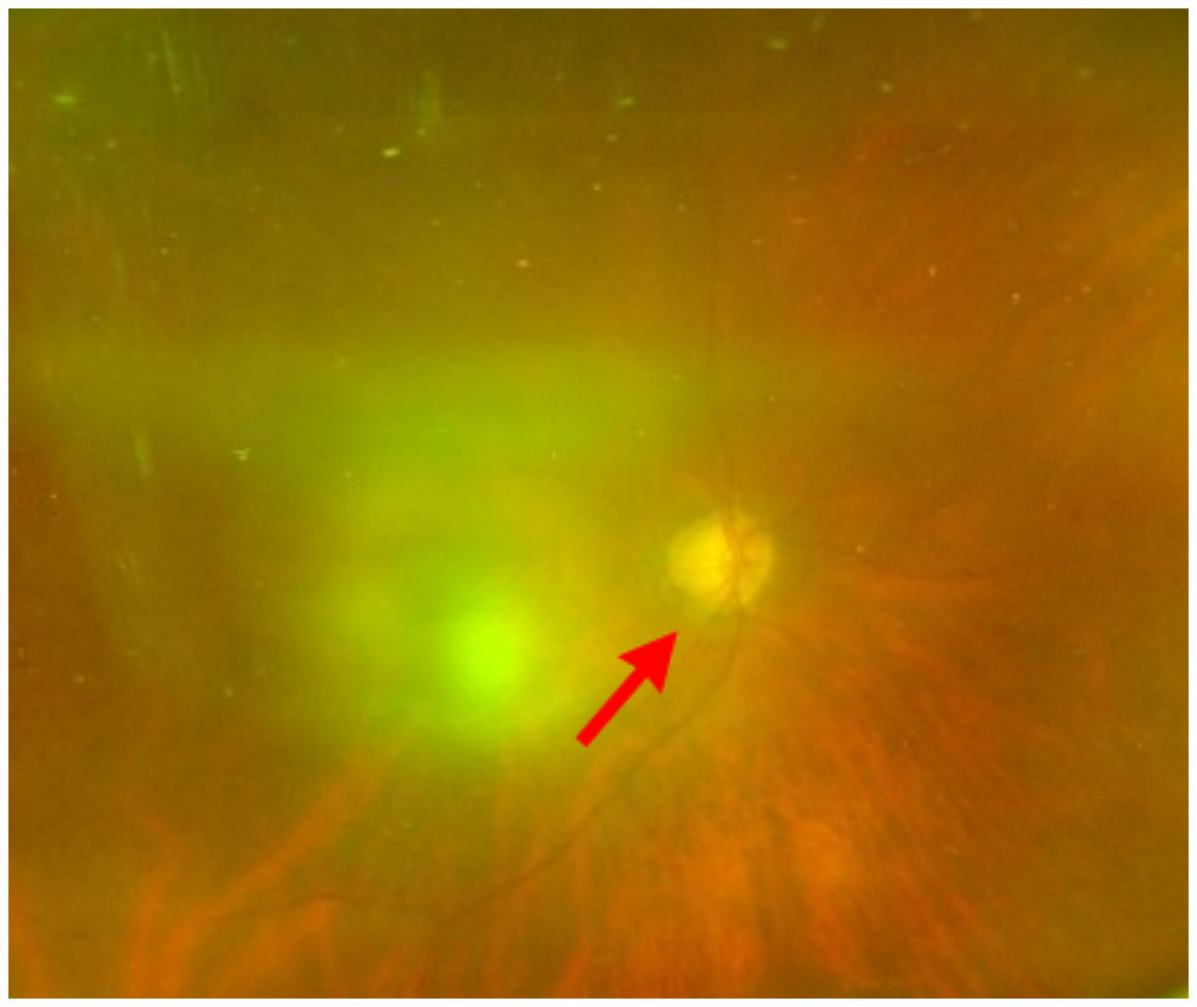
Postoperative fundus photograph of the right eye. The pale optic disc is indicated by the red arrow.

On postoperative day 1, BCVA in the right eye was hand motion and IOP was 14 mmHg. Slit-lamp examination showed mild corneal edema, a well-formed anterior chamber without exudation, and a well-centered IOL. Fundus examination revealed a pale optic disc (Figure 3).

At 1 week postoperatively, BCVA improved to finger count at 30 cm, IOP was 16 mmHg, the cornea was clear, the anterior chamber remained deep and quiet, the IOL was stable, and the optic disc was still pale. At 1 month postoperatively, BCVA reached 0.08 and IOP was 13 mmHg. Clear cornea, normal anterior chamber depth and transparent aqueous humor were noted. The IOL maintained good position, and the optic disc remained pale.

## Discussion

Blunt ocular trauma has highly variable clinical presentations and may affect multiple anterior and posterior segment structures. Isolated posterior capsule rupture without anterior capsule involvement is extremely rare, with only a small number of case reports in the literature (2–5).

The pathogenesis of this distinctive injury can be explained by anatomical and biomechanical properties. The anterior and posterior lens capsules differ markedly in thickness. The anterior capsule thickens with age and exhibits greater mechanical strength, whereas the posterior capsule is thin and remains stable in thickness throughout life (6)^.^ Biomechanical studies indicate that stress distribution during blunt force transmission is significantly higher in the posterior capsule, making it more susceptible to rupture (7). In this patient, low-velocity indirect blunt force caused rapid equatorial distension, resulting in focal rupture of the thin posterior capsule. Zonular injury is a common accompaniment of blunt lens trauma. In elderly patients, physiological weakening and age-related degeneration of zonular fibers reduce mechanical resistance, leading to widespread dehiscence even after mild injury (8)^.^ Intraoperative findings confirmed zonular loss from 6 to 9 o’clock, consistent with the typical pattern of blunt ocular trauma in aged eyes.

The hallmark feature of this case was complete expulsion of the lens nucleus and cortex from the capsular bag into the vitreous cavity, creating an “empty capsular bag” appearance. Previous reports mostly described partial cortical loss or residual lens material, with very few cases of complete posterior dislocation (2–5). This complete evacuation is strongly associated with advanced age, which leads to extensive cortical liquefaction and increased fluidity (9). In contrast, most reported cases involve children or young adults with gel-like, viscous cortex that does not readily evacuate even after posterior capsular rupture. In our patient, rapid escape of liquefied cortex after posterior capsule rupture led to complete posterior lens dislocation, compounding the clinical complexity. Consequently, a thorough preoperative assessment was conducted.

Preoperative slit-lamp examination confirmed an intact anterior capsule. However, due to inadequate pupillary dilation, the status of the peripheral zonular fibers could not be fully evaluated. Additionally, vitreous haze and a poorly visible dislocated lens nucleus further limited comprehensive fundus evaluation. Therefore, preoperative multimodal imaging—including B-scan ultrasonography, UBM, AS-OCT—was performed to obtain supplementary information (10–13). These imaging modalities confirmed the presence of an intact anterior capsule, posterior capsule rupture, complete lens dislocation into the vitreous cavity, and zonular dehiscence, thereby supporting the clinical diagnosis.

Management of posterior capsule rupture depends on tear size and location, vitreous prolapse, and degree of lens dislocation. In elderly patients with complete posterior lens dislocation, retained lens fragments and persistent vitreoretinal traction markedly increase the risks of retinal tears, retinal detachment and cystoid macular edema. Therefore, PPV combined with total lensectomy serves as the first-line treatment for patients presenting with complete lens dislocation and extensive zonular dehiscence (14). This surgical approach can remove dislocated lens contents, release vitreoretinal traction, and further reduce the incidence of these vision-threatening retinal complications. In elderly patients with multiple zonular dehiscence and total loss of capsular support, conventional in-the-bag IOL implantation is not feasible. Scleral-fixated IOL suspension offers excellent intraoperative control and long-term stability, making it the preferred approach for such complex cases (14). We formulated an individualized surgical plan for the patient. The operation was uneventful, and the anatomical structure remained stable in the early postoperative period. It should be noted that ciliary sulcus fixation, which is less invasive, is a reasonable alternative when adequate zonular support is preserved. In this case, zonular dehiscence involved approximately 3 clock hours (from 6 to 9 o’clock). Intraoperatively, the remaining zonular fibers, though not frankly ruptured, appeared markedly lax and attenuated. Despite an intact anterior capsule, the overall zonular support was deemed insufficient for stable ciliary sulcus fixation, which would have carried a high risk of late IOL dislocation. Elderly patients have fragile ocular tissues. Once IOL dislocation occurs, reoperation will bring substantial trauma and risks. Alternative strategies, including staged surgery, delayed secondary IOL implantation, or temporary aphakia, were considered. However, they were deemed less suitable due to the patient’s advanced age, limited tolerance of multiple interventions, and higher risk of complications from prolonged aphakia. After comprehensive evaluation, we ultimately performed scleral-fixated IOL implantation to achieve favorable long-term outcomes. Nevertheless, this technique has several limitations. Its potential complications mainly include suture erosion, IOL tilt, late dislocation and endophthalmitis (14). A large cohort study involving 103 eyes undergoing scleral-fixated IOL implantation reported postoperative complications: transient intraocular pressure elevation (7.8%), cystoid macular edema (6.8%), vitreous hemorrhage (1.9%), and IOL tilt or subluxation (2.9%) (15). Due to age-related tissue fragility in elderly individuals, the risks of suture breakage and late IOL dislocation are further increased, and the disability risk related to reoperation is significantly higher in this population. In conclusion, although scleral fixation provides reliable long-term stability for this patient, clinical decision-making requires a careful balance between anatomical indications and the risks of long-term complications.

Notably, pale optic disc was observed in the affected eye during and after surgery. Preoperative UBM ruled out angle recession and narrow anterior chamber angle, so secondary glaucoma induced by traumatic angle recession and chronic angle-closure glaucoma were largely excluded. Preoperative opacity of the refractive media prevented visual field and OCT examinations. Postoperatively, further examinations were declined by the patient’s family due to advanced age. Consequently, there was a lack of objective evidence for optic nerve status, and the exact etiology of pale optic disc could not be determined for the time being. After excluding glaucoma associated with anterior chamber angle abnormalities, the possible explanations include age-related optic nerve degeneration (16), traumatic optic neuropathy (17), ischemic optic neuropathy, or a combination of multiple factors. This case has certain limitations. Objective evaluations of the structure and function of the optic nerve were not performed either preoperatively or postoperatively. This suggests that for elderly patients with ocular blunt trauma complicated by refractive media opacity, clinicians should complete optic nerve-related examinations as early as possible to identify concurrent lesions and provide reasonable visual prognosis for patients and their families.

## Conclusion

This case of posterior capsule rupture with complete posterior lens dislocation following blunt trauma in a 90-year-old patient expands the clinical data and phenotypic spectrum of this extremely rare ocular injury. Such cases lack typical signs and are easily misdiagnosed or missed. Multimodal imaging aids in accurate injury classification. Individualized surgical plans should be tailored to patient age, baseline ocular conditions, and severity of tissue damage. In elderly patients, special attention must be paid to concomitant occult optic neuropathy to optimize clinical outcomes.

## Data Availability

The original contributions presented in the study are included in the article/supplementary material, further inquiries can be directed to the corresponding author.
